# Why Music Therapists Choose to Work with a Clinical Population: An International Pilot Survey

**DOI:** 10.3390/ijerph19159463

**Published:** 2022-08-02

**Authors:** Avi Gilboa, Chava Wiess, Ayelet Dassa, Melissa-Mercadal Brotons, Eva Frank-Bleckwedel, Elisabeth Kaczynski, Jiri Kantor, Beate Roelcke, Patricia Sabbatella

**Affiliations:** 1 Music Department, Bar-Ilan University, Ramat-Gan 5290002, Israel; 2 Arts Therapy Department, Music Therapy Program, David Yellin College of Education, Jerusalem 9434518, Israel; chavaw@dyellin.ac.il; 3 Escola Superior de Música de Catalunya, Universitat Ramon Llull, 08013 Barcelona, Spain; brotons@compuserve.com; 4 Institut für Musiktherapie, Hochschule für Musik und Theater Hamburg, 20148 Hamburg, Germany; eva.bleckwedel@hfmt-hamburg.de; 5 EMTC Delegate of Austria, 1120 Vienna, Austria; ek@oebm.org; 6 Center of Evidence-Based Education and Arts Therapies: A JBI Affiliated Group, Institute of Special Education, Sciences, Faculty of Education, Palacký University Olomouc, 779 00 Olomouc, Czech Republic; jiri.kantor@upol.cz; 7 Department of Music, Zurich University of the Arts, 8005 Zürich, Switzerland; beate.roelcke@zhdk.ch; 8 Faculty of Sciences Education, University of Cádiz, 11519 Cádiz, Spain; laboratorio.musicoterapia@uca.es

**Keywords:** music therapy, population choice, clientele, online survey, gender differences, professional identity

## Abstract

(1) Background: Throughout their career, music therapists make decisions regarding the clinical population they choose to work with. Though such decisions can have broad implications on the professional development of the music therapist, not much is known about the reasons for making these decisions and whether they are affected by demographic or professional factors. (2) Methods: In this pilot study, we surveyed 439 music therapists from six countries (i.e., Austria, the Czech Republic, Germany, Israel, Spain, and Switzerland) using an online questionnaire. We asked the respondents to explain why they chose to work with their main clienteles, and we examined whether their reasons were connected to demographic factors such as country of origin, gender, and seniority, and professional factors such as experience as a music therapist and population one works with. (3) Results: The category analysis of these responses pointed at nine distinct reasons that could be grouped into “practical reasons”, “reasons of connection”, and “innovation”. There were differences in reasoning between music therapists from different countries, and with different degrees of seniority, but not between male and female music therapists. (4) Discussion: The implications on training programs and on policy makers are discussed as well as the importance of this subject to the development of music therapists’ professional identity.

## 1. Introduction

Music therapy is used with a growing variety of clinical populations (see just as an example the following books that refer to a large variety of clinical populations [1,2,3,4] and more). This is reflected in a growing variety of courses and placements in training programs around the world [4,5,6]. After graduating, music therapists then have to make pivotal decisions regarding the clinical population they choose to work with. Our personal experience as educators shows that for some graduates, the decision is clear, and is connected to the initial experience they gained during the training. For others, the decision is connected to a long-lasting dream to work with a certain population. Yet, for others, there is no real choice—in some countries, working opportunities as music therapists are scarce and so they start working at whatever job is available. The choice to work with another clinical population might reoccur throughout one’s career, and with each transition, considerations regarding the clinical population can emerge.

We believe that the choice to work with one clientele or another is part of the overall developmental process that music therapists undergo and that it might have an effect on defining and maintaining their professional identity. This process begins as early as the initial choice to become a music therapist [7,8], continues in the early stage of training [1], and is thereafter important for the music therapist’s healthy development [9,10].

Other professions have studied such choices and found them to be very important for professional development. Ref. [11], for instance, examined how social work students explained their choice of population. They found that some of them chose the clientele according to practical reasons such as better working conditions, while others referred to reasons such as their quest for personal development and satisfaction. Some of the respondents had prior experience with specific populations, and they had a natural pull to work with that population. This study also found that students who worked with the elderly as part of their field work tended to continue with this population after they graduated (see [12,13] and others who have researched this in the field of social work). Other studies that focused on psychotherapists’ choices, [14], for instance, found that psychotherapists who work with sexually abused children explained their choice as resulting from a great satisfaction and feelings of fulfilment when working with these children (see [15,16] and others who have researched this in the field of psychotherapy).

In contrast to the interest that other professions have given to this subject, only scant research has focused on music therapists’ considerations and rationalizations for working with one clinical population or another. Dassa interviewed four music therapists who chose to work with Alzheimer’s disease (AD) clients, and she found that while some of the reasons for choosing to work with this population were connected to their personal inclinations, others were connected to values and beliefs regarding what music therapy is about and what it can do [17]. For instance, participants mentioned having a positive acquaintance with elderly people in the past (including during their internship), which prompted them to work with this population, and enabled a good relationship with them. For some participants, there was a connection between the therapists’ musical culture and background and their clients’ musical styles, which were typically “old-fashioned”. It also emerged that the choice to work with AD clients was associated with some of the music therapists’ personality traits, such as a relaxed temperament and a need for order and organization. Finally, the choice was linked to the conditions at the workplace and the need for comfort, satisfaction at work, and the possibility of developing and seeking out new challenges [17].

Ref. [18] studied the music therapists’ “choice narrative” when choosing to work with different clinical populations. Her assumption was that such choices were deeply connected to personal and professional identity and that these were continuously built and rebuilt according to the music therapists’ narrative about themselves [19,20]. Yona-Blechman’s analysis of interviews with four experienced music therapists led to several clusters of reasons for choosing to work with specific clinical populations: (1) the choice was connected to the work environment and to proper work conditions, such as a suitable salary and an appropriate music therapy room. Participants stressed that the team and a working environment that could contribute to professional development was important; (2) the choice was connected to positive feelings towards the population, such as feeling comfortable with the clinical population, having a personal connection with that specific population, and a sense of satisfaction and joy when working with this population and being challenged by the work; (3) the choice was connected to personal traits that were required for working with that specific population (e.g., being patient and therefore wanting to work with a population that requires a lot of patience, or being a rebellious type and therefore being attracted to adolescents with mental health issues); (4) the choice of population was connected to one’s professional identity such that the work with the chosen population enabled the music therapists to fulfil their clinical approach and values; and (5) the choice was connected to one’s personal and family history such as being connected to a mentally ill family member and then being inclined to working with similar clients.

In the present study, we aimed to further understand the reasons underlying music therapists’ choices to work with different clinical populations and to see whether and how these choices are different for music therapists from different backgrounds. This subject can have important implications for music therapy students and graduates, as well as more experienced music therapists who are continuously searching for their professional identity. It can also help training programs to understand the potential implications of exposing students to different client populations. To date, this subject has only been examined in a few studies and with a limited number of participants and countries. To gain a broader scope on this subject, we initiated an international pilot study that covered six countries: Austria, the Czech Republic, Germany, Israel, Spain, and Switzerland. Though these countries were not in any way a representative sample of countries in which music therapy is actively practiced, it served as a good starting point for a pilot study, and it could provide general findings and directions for a further inquiry in a bigger sample of countries. There was however a reasonable diversity between the countries that were chosen for this study that referred to: (1) music therapy being in different phases of development (e.g., in the Czech Republic it is in an initial phase of development while in Austria and Switzerland, musical therapy is officially and legally recognized as a profession); (2) music therapy being taught at different academic levels (e.g., in Israel—MA only, in Austria—both BA and MA); and (3) music therapy being practiced with a variety of populations using an assortment of approaches.

The data of the current study were collected as part of a longer online survey that addressed a broad array of subjects relevant to music therapists, such as the satisfaction with different aspects of work, perceptions on burning issues in music therapy, and other profession-related subjects. The full survey included questions that were partly open-ended and partly closed, using a mixed-methods framework (i.e., analyzing the open-ended questions qualitatively and the closed questions quantitatively). For the current study, only questions that referred to the choice of clientele and relevant demographic information were analyzed. As in other international surveys conducted among music therapists, the online platform enabled a good outreach to participants and an efficient way to collect data (see [21,22,23,24] for similar uses of online surveys for music therapists on a national and international scale). Specifically, in this study, we referred to and analyzed questions in which music therapists explained why they chose to work with the clinical populations they treated. We also analyzed demographic information about participants, such as gender, years of experience, and population they currently worked with to see whether there were connections between the reasons they gave and their background information. This was a Triangulation Design: Data Transformation Model in which the qualitative data (QUAL) was quantified (QUAN) [25]. Specifically, for the present study, the answers to the open-ended question referring to the reasons for choosing a clientele were qualitatively analyzed to form categories. Once the categories were formed, we then counted the number of participants that chose each category and analyzed these numbers quantitatively with descriptive statistics. The research questions were explorative:

1. How do music therapists explain their choice to work with different clinical populations?
2. Are the reasons for working with client populations connected to different demographic factors such as country of origin and gender, and professional factors such as experience as a music therapist and population one works with? If so, in what ways?

## 2. Materials and Methods

Ethical Considerations: The study was approved by the Ethics Committee of the music department at Bar-Ilan University (E.MUS.2021-13). Participants took part in the study knowing that their anonymity was guaranteed. 

Participants: The inclusion criterion for the survey was that respondents were active music therapists. In Austria and Switzerland, being an active music therapist automatically implied being registered in the professional association. So, in these countries, this was also an inclusion criterion. In the Czech Republic, where music therapy is differently defined as a profession, participants were included as long as they had some level of qualification in music therapy and practiced it, even if their primary profession was different, such as speech therapy and clinical psychology. Practicing students and nonactive music therapists were not included in the survey. A total of 439 music therapists (348 females and 91 males) from 6 countries participated in the study: Austria (n = 24), the Czech Republic (n = 64), Germany (n = 83), Israel (n = 138), Spain (n = 53), and Switzerland (n = 77). Their mean age was 46.0 (SD = 11.6) and they had a mean 12.7 years of experience (SD = 9.8) as music therapists. A total of 312 respondents reported that they worked with children, 107 with adolescents, 198 with adults, and 157 with elderly people.

Tools: As part of a larger international study, we created a questionnaire that included issues that were of interest to the researchers and understudied by previous researchers. The aim of this study was to understand the attitudes of music therapists from different countries and different clinical orientations about various subjects such as satisfaction with different aspects of work, perceptions on burning issues in music therapy, and other profession-related subjects. We converted the questionnaire into an online survey. For the present study, we focused on demographic questions regarding country of origin, gender, number of years working as a music therapist, the main clinical populations they worked with, and the reasons they chose to work with those populations (open-ended question).

Procedure: The online survey was distributed simultaneously among music therapists in the six selected countries, between March and May 2020. Participants answered the survey voluntarily and anonymously via Google Forms, and in Switzerland, via a Lime survey. After data collection ended, we aggregated the data from all countries and compiled an SPSS file for analysis. The questions regarding choice of clinical population were first worked into categories using data-driven qualitative categorization based on [26]. Three of the authors first read through all answers several times to obtain a general sense of the data and then, independently, looked for recurring answers and possible categories. They then compared their independent categories, negotiated the differences, and combined them into an agreed-upon codebook. Using this codebook, they sampled 100 responses and coded them independently. Again, differences were negotiated, and adjustments to the codebook were made whenever there were too many discrepancies between the coders. Using this refined codebook, the researchers coded the rest of the sample, obtaining sufficient interjudge reliability (90% of the cases were exactly the same for all coders). 

After crystalizing the categories, a quantitative analysis was conducted in which we counted the number of responses in each of the categories. These data, as well as demographic questions that were answered numerically, were analyzed using descriptive statistics procedures based on absolute and relative frequencies. Because of the exploratory nature of this study, descriptive statistics methods, and not inferential statistics, were considered sufficient for the purpose of identifying major trends. Therefore, no statistical tests were conducted, and no *p* values calculated. This also implies that the results refer to the specific sample of this study and do not generalize to the broad population of music therapists.

## 3. Results

To examine how music therapists in this study explained their choice to work with different clinical populations (first research question), we gathered all of the reasons that participants gave for choosing to work with their clients. We performed a qualitative category analysis of these reasons as explained above in the “procedure” section and came up with three main categories: practical reasons (P), reasons referring to connection (C), and reasons pointing at innovation (I) (see Table 1). 

These categories then divided into nine subcategories: practical reasons for choosing a clientele, pertained to the job having convenient conditions, such as being close to home, or paying a good salary (this was denoted as P1); the job being available such as a colleague recommending it, or a job opening that was available at the time (denoted as P2); the job being available as a continuation of field work or internship (denoted as P3); and continuing as a music therapist at a workplace where the participant previously worked in a different capacity (e.g., a nurse, a teacher; denoted as P4). See the rightmost column of Table 1 for examples of typical reasons that were given in participants’ open-ended answers.

The second category we found pertained to a deep connection that participants expressed towards a specific clinical population. There were four kinds of these connections: music therapists developed a deep interest of and curiosity towards the clientele, and wanted to deepen the connection (denoted as C1); music therapists felt emotionally connected to the clientele, expressing that they loved working with them, and that they could really feel the clientele and their needs (denoted as C2); music therapists referred to the clientele’s special connection to music and the special role music had in helping them (denoted as C3); and music therapists referred to a former personal connection with this clientele, usually a family member with a similar disability, or a friend that inspired them earlier in their lives to work with this clientele (denoted as C4).

The third category we found pertained to an innovative stance that the music therapists expressed. In this category, which did not have subcategories, music therapists referred to the importance of working with an underprivileged population or with populations that were neglected to some degree or another. They felt it was their mission to work with this population and to promote music therapy with them, and that if they did not take the lead, nobody else would.

In Table 2, we present the percentage of times each of these categories (P, C, and I) and subcategories (P1, P2, P3, P4, C1, C2, C3, C4, and I) were mentioned by the participants. Results are presented for the entire sample (rightmost column) and separately for each of the six countries that participated in the survey.

Looking at the rightmost column (entire sample), one can see that the most common reasons for working with clientele were in the connection (C) category (53% of the answers), followed by practical reasons (P, 42%). Within these categories, “job opening” (P2) was the most prevalent technical reason (22% of the answers), and emotional connection (C2) was the most frequent answer in the connection category (27%). The least dominant reasons were “convenience” (P1, 2%), “connection to music” (C3, 4%), and “innovation” (I, 4%).

To answer the second research question (“are the reasons for working with client populations connected to different factors such as country of origin, gender, experience as a music therapist and population one works with?”), we conducted several comparisons. First, we counted the reasons for choosing clientele, separately for each country (see Table 2). As can be seen, different countries had different distributions of reasons. Referring to the general categories P, C, and I, in Austria, the Czech Republic, Spain, and Switzerland, about 50% of the reasons were technical and about 45% were related to connection, while in Germany and in Israel, about 60% were related to connection and only about 35% were technical reasons. The innovation category was highest in the Czech Republic (10% of the answers) and lowest in Switzerland (1% of the answers). An examination of the subcategories revealed several findings. The first was that “internship” was a dominant reason in some countries (17% in Austria, 14% in Israel) but not in others (0% in the Czech Republic, 2% in Germany). Second, that “transfer from another profession” was quite predominant in some countries (22% in the Czech Republic, 14% in Spain and in Switzerland) but not in others (5% or less in Austria, Germany, and Israel). In addition, in Germany “connection through interest” was more prevalent than in other countries (26%), in Israel “emotional connection” was highest (38%), and in the Czech Republic, “innovation” (10%) was more common than in other countries.

In Table 3, we compare female and male music therapists with regards to the reasons they provided for choosing the clientele they work with.

Generally speaking, one can see that results are very similar for female and male music therapists. The only difference worth noting is that female music therapists referred more frequently to “emotional connection” than male music therapists (29% vs. 19%, respectively), while male music therapists referred more prevalently to “connection through interest” than female music therapists (24% vs. 14%, respectively). 

We then compared music therapists’ reasons for working with clientele according to their years of experience as music therapists (see Table 4). We divided the sample according to the 33rd and 67th percentile cut-points: less than or equal to 6 years of experience, whom we labelled as novel music therapists (NMT), more than 6 and less than 15 years of experience, whom we labeled as experienced music therapist (EMT), and more than 15 years of experience, whom we labeled as very experienced music therapists (VEMT).

The data indicate that in most cases the answers are similar among the groups. In fact, NMTs and EMTs seemed to have approximately the same answer patterns, with practical reasons (P category) adding to 49% (for NMTs) or 47% (for EMTs) of the answers, and connection-related reasons (C category) adding to 47% of the answers (for NMTs) or 49% (for EMTs). The main difference between the groups can be observed in regard to the VEMTs, who exhibited lower rates of practical reasons (P category; 31%) and higher rates of connections-related reasons (C category; 64%). An examination of the subcategories showed that these differences were the result of the difference in the following subcategories: VEMTs reported a much higher incidence of choosing clientele because of interest (25% for VEMTs vs. 11% for NMTs, and 13% for EMTs); VEMTs reported a much lower incidence of choosing clientele as a result of continuing an internship (4% vs. 13% for NMTs and 10% for EMTs) or transferring from another profession (3% vs. 12% for NMTs and 10% for EMTs).

We next examined whether reasons for choosing clientele were connected to the actual clientele the participants worked with. We divided the clientele according to age group, differentiating between children (including babies and toddlers), adolescents, adults, and elderly people (see Table 5). The total section of Table 5 shows that an adult clientele was chosen by participants for practical reasons (P category) more than any other age group (50% for adults compared to 42% for children, 41% for elderly people, and only 33% for adolescents). In contrast, an adolescent clientele was chosen because of a feeling of connection (C category) more than any other age group (63% for adolescents compared to 54% for elderly people, 52% for children and 46% for adults). In reference to the more detailed subcategories, it could be seen that while some subcategories were similar across age groups (P1—convenience: between 1% and 4%; C1—connection out of interest: between 13% and 18%; C4—personal connection: between 4% and 7%; and I—Innovation: between 4–5%), some subcategories were quite variable across age groups. Emotional connection (C2 subcategory), for instance, was frequently mentioned as the reason for choosing an adolescent clientele (38%) but much less so when choosing an adult clientele (20%). Job opening (P2 subcategory) was more frequently mentioned as the reason for choosing elderly people (28%) but less so when choosing an adolescent clientele (19%).

## 4. Discussion

The first aim of this study was to get an initial understanding of music therapists’ reasons for choosing to work with a clientele. Previous answers to this question [17,18]. were based on very small samples of music therapists from one country, and here we aimed to get a broader scope based on a larger sample obtained from different countries.

Results show that there are three categories of reasons for choosing to work with one clientele or another: practical reasons (the P category), reasons of connection with the clientele (the C category), and reasons of innovation (the I category). We believe these categories reflect forces that impact music therapists in different ways when facing the question of which clientele they choose to work with. The two big categories (P—42% and C—53%) seem to represent reasons that come from different, perhaps even opposing, directions. On the one hand, practical reasons represent what the environment offers. The better the conditions in a job with a specific clientele are, or the more available or familiar the job is to the music therapist, the more it will “pull” the music therapist to work with that clientele. On the other hand, reasons of connection represent internal ideas and fantasies that the music therapist holds about different clinical populations. The more specific and positive these ideas and fantasies are, the more they will “push” the music therapist to seek work with that clientele, even if this job’s conditions are less favorable. These forces are similar to and echo the division that is often made in the career choice literature between pecuniary (e.g., high paid salary) and nonpecuniary (e.g., identity-related issues) reasons in job seeking (e.g., [27,28]). The novelty in the present study is that the internal and external factors may not only affect career choice in general (e.g., choosing to train as a music therapist over other possible professions), but also microdecisions that are made within the profession (e.g., choosing to work with one clientele or another).

The “Innovation” category (typically explained as follows: “I chose this population because I felt it was totally neglected and that it deserves more clinical attention”) was smaller than the two others (I—4%), but still, we believe it is worth noting. Music therapists who give such reasons are special in that they dedicate their efforts to unattended, often underprivileged populations. Typically, there will be nothing practical about working in these places: the music therapy position will be poorly budgeted (if at all), the music therapy room will perhaps be underequipped (if at all), and there may not be other music therapists around to consult with, nor will the colleagues in this place necessarily understand the essence and importance of music therapy for this clinical population. According to their answers, the “innovator” music therapists pioneer new clinical populations that have not yet been treated by music therapists in their country. In doing so, they help to redefine and broaden the scope of clinical populations in their country. Although this category was small, it seems important for the development of a profession and therefore deserves further research.

An examination of the more specific subcategories we found showed that some resembled what was found in previous studies. For instance, feeling connection with a clientele was found as a reason for choosing a clientele in both [17] and [18]. In the present study, if we include the C2 subcategory (emotional connection to the specific population) that was the most prevalent subcategory (27%) and the C1 subcategory (connection out of interest; 16%), we can conclude that a connection to the clientele is definitely a central reason for music therapists to choose to work with a particular clientele. C4 (i.e., having a previous connection with the clientele) is another subcategory that was found in the present study that supports findings from previous studies. This reason was found in [18] and [17] as well as in studies that examined the reasons people chose to work as psychotherapists (e.g., [29,30]).

Some reasons, however, were novel to the present study and were not found in previous studies. For instance, choosing a clientele because of a mere job opening (subcategory P2, 22%), or as a continuation from another profession (subcategory P4, 9%) were not found by [17] or by [18]. We believe that this is because the present study addressed music therapists from various contexts, cultures, and countries where music therapy as a profession is at different stages of development, while the previous studies focused on very small samples of Israeli music therapists. Although in other countries it is common to transfer to music therapy from another profession while remaining in that profession ([31], for example), often because there is no basic music therapy education, in Israel it is less common, which could explain why this was mentioned as a reason for choosing a specific clientele in the present study but not in the Israeli studies. In addition, the present study included a considerable number of inexperienced music therapists, which might explain why “job opening” was mentioned as a reason. In the Israeli studies, on the other hand, inexperienced participants were the exception. Most of them did not take the first job opening they found, which might explain why they did not refer to it as a reason for choosing to work with their clientele. 

Another reason that was novel to the present study was “continuation of the internship” (subcategory P3, 9%). This reason points at the importance of internships in shaping the future decisions of music therapists, especially with regards to specialization with different clinical populations. The initial internship experiences often serve as a gateway to the professional world, defining the choice of clientele. Training programs should keep this in mind when deciding how much emphasis to put on the process of choosing the internship placement and their degree of involvement in it. This is especially relevant within the context of this international survey because programs in different countries treat internship differently (e.g., the number of required internship hours can vary from country to country; in some countries the students find their own placement and the training program approves, while in other countries, the training programs select the placements). Finding the P3 subcategory in this study can work as an encouragement for decision-makers to put as much emphasis as they can on the internship as it can have a substantial effect on interns’ future decisions. 

All in all, the fact that this study yielded a broad array of reasons for choosing to work with a clientele and the fact that some of them were novel, reinforces the need for this study and for further studies that can expand and refine an understanding of this topic.

The second aim of this study was to determine whether the reasons for working with a clinical population were connected to demographic (country of origin and gender) and professional variables (experience as a music therapist and clinical population that the music therapist actually works with). The analyses we conducted yielded some interesting results, but these results must be taken very carefully because of the descriptive nature of the analyses and because of the small samples in some of the countries. We will therefore suggest tentative explanations for the results, and strongly recommend that these explanations are further examined in future studies.

Regarding the country of origin, we found discrepancies in the reasons given by music therapists from different countries. Possibly these differences can be explained by the fact that the music therapy profession, in the countries participating in this study, developed under different conditions, some of which recognize music therapy more than others, and some of which have more experience than others. There are differences on many levels such as: (1) when the first training courses opened (in Austria—in the late 1950s, in Germany in the 1970s, in Switzerland and in Israel—in the 1980s, in Spain—in the 1990s, and in the Czech Republic—in 2019); (2) what the academic level of the training programs is (in Spain, Israel, and the Czech Republic—master’s level only; in Switzerland—master’s and nonacademic levels; in Austria—bachelor’s and master’s level; and in Germany—bachelor’s, master’s, and nonacademic levels); and (3) whether music therapy is legally recognized in the country (in Austria and Switzerland but not in the rest of the countries in this study). 

With regards to the specific countries in the present study, the Czech Republic represents a country that is relatively new to the profession, which may explain why it was low on the “internship” reason (P3): since the academic-based programs are still new in this country, there are rarely any internship placements to acquire one’s affinity to work with a certain clientele. The novelty of the Czech Republic may also explain the high incidence of the “innovation” reason (I): because the variety of clinical populations is still low in this country, there are more music therapists seeking to work with new, unattended populations. The higher rate of connection reasons (the C category) in Israel and in Germany, might be due to the wealth of work opportunities that exist in these countries, which makes it possible to choose clinical populations according to connection (the C category) and less according to practicalities (the P category). The higher incidence of the “internship” reason (P3) in some countries (e.g., Austria, Israel) may be due to the fact that placements in these countries are more highly regulated by the training programs than in other countries. Clearly, music therapy in different countries has different histories, and different factors that influence its development, which international studies have recently begun to outline (e.g., [21,32]). The current study points at the value and the need to continue this examination. 

A comparison between genders did not yield too many differences. Male and female music therapists gave similar reasons for choosing their clinical populations and the distribution of these reasons was about the same. The exception to this were the C1 subcategory (connection out of interest), where men featured more prominently than women and the C2 subcategory (emotional connection), where women featured more prominently than men. Although these differences are consistent with the masculine (more logical) and the feminine (more emotional) stereotypes, they may also be explained as representing discrepancies in the ways of expressing ideas between men (i.e., expressing a connection to clients as an “interest” in them) and women (i.e., expressing a connection to clients as good feelings), but not differences in their actual desire for connection with that clientele. In any case, obtaining similar results among male and female music therapists is consistent with other studies that compared male and female music therapists and did not find significant differences. For instance, [33] reviewed burnout among music therapists and did not find gender differences, and [34] examined the abilities of music therapists to recognize emotions expressed in improvisations and did not find gender differences (see also [35], who showed no gender differences among graduating music therapists in how they equipped an imaginary music therapy room).

The very experienced music therapists (VEMT) appeared to provide different answers than the experienced (EMT) and novel music therapists (NMT). Most notable was that they mentioned many more “connection” reasons (C category) and many fewer practical reasons (the P category) in comparison to the less experienced music therapists. These results are reasonable, considering that as music therapists gain more experience and expertise, they are exposed to a greater range of opportunities, which enables them to choose their clientele according to their inner interests, and less out of practical reasons. This explanation clearly needs validation in further research.

Music therapists working with adolescents tended to choose their clientele out of connection to their clients (the C category), especially emotional connection (C2), more than music therapists working with any other age group. Respectively, they tended to choose their clientele for practical reasons (the P category), especially because of job opening (P2), less than music therapists working with other age groups. This could be explained by the fact that in most countries, work with adolescents is not yet as prevalent as the more traditional work with children and adults. Thus, fewer job openings or internship spots are available, and those who do eventually work with adolescents, do this against the natural “flow”, possibly because they feel an emotional connection to their clients. If this is indeed the case with adolescents, it is questionable why we do not see similar results regarding elderly clientele, which is also considered a relatively new clientele in music therapy. However, it could be that there are other factors interacting with the music therapists’ decisions for choosing this clientele, which require further examination. 

### Research Limitations and Suggestions for Future Research

There were several limitations in this study. First, sampling was neither random nor representative, which implies that findings and conclusions must be considered very carefully and speculatively. Particularly in Austria, the response rate was only about 6% and so a very small portion of music therapists in Austria was actually represented in this study. Second, the variety of countries was small, only partially representative of European countries. It is, therefore, recommended to initiate a study on this subject in a greater number of countries, not only in Europe, and thus to get a fuller understanding of this subject. Third, the classification of the types of clienteles was limited. This is because there are different aspects according to which one can categorize clientele (e.g., type of disability, type of treatment, age group of clients, and more), which results in subgroups that are not necessarily mutually exclusive. This issue was brought up in previous studies and dealt with in different ways [21], but there is still no agreed-upon parsimonious way to categorize clinical populations. In this study, we chose the simpler age group division, which is in itself important, but certainly not sufficient in representing the complexities of different clinical populations. Finally, the categorization process of different reasons for choosing clientele, although thorough and systematic, was based on subjective accounts of three of the authors of this study. It is therefore important, after collecting more data on this subject, to reattempt categorization, compare it with the present categorization, and refine and broaden it.

We suggest that further studies should be conducted to broaden and re-examine the findings of this study. First, as we found that internships are important in the professional direction of music therapists, it is recommended to focus on this topic and to see how the different countries’ approaches to internship shape different possible connections among beginning music therapists and their evolving professional identity. An examination of these factors may show that there are approaches to internship that yield better conditions for professional development. Second, when more closely examining the reasoning process of music therapists when choosing to work with a specific clientele, it is important not only to ask them which clienteles they have chosen to work with but also to ask about clienteles that they may have avoided. Sometimes, such “reverse” information can add a lot of insight and understanding. For instance, stating that one would prefer not to work with elderly people (which is something trainers in different training programs may frequently hear), and trying to understand why not, can provide complementary information not only about what attracts, but also about what repels. A similar thread of research can examine how music therapists deal with the challenges and difficulties they face with their clientele, and this too, can be very enriching and useful for music therapists worldwide. Finally, we believe that further research is required in order to understand the professional identities of music therapists and how they evolve and develop. Different models for understanding professional identity were offered with regards to other professions (e.g., [36]), but only initial attempts have been made to connect them to music therapy (e.g., [9], 2016 regarding art therapists).

## 5. Conclusions

Through the lens of a very specific choice that music therapists make several times in their career, namely, the choice of a clientele to work with, some very interesting identity aspects of our profession and its development were exposed. We found that choosing a clientele can come from practical reasons but also from a deep emotional connection to that clientele. We found that some music therapists choose their clientele because they want to innovate and to initiate social change, and they might have an impact on the development and formation of the profession in their country. We also point at the possibility that different countries offer different conditions to music therapists, and thus form unique music therapy environments that subsequently affect the music therapists’ abilities to choose their clientele and develop their professional identities. One such factor, which is applied differently in various countries, is the internship in training programs that serves as a gateway to professional life. Finally, we point at the possibility that there is a professional development path that provides more experienced music therapists with greater freedom in choosing their clinical populations. This exploratory study provided a preliminary glimpse into an understudied subject. We believe that more efforts should be made to understand the choices that music therapists make throughout their career and to subsequently gain a more in-depth understanding of the issues of professional identity and professional development.

## Figures and Tables

**Table 1:** Reasons for working with clinical populations—categories, subcategories, and typical answers.

Category	Subcategory		Typical Reasons (Quotations from Survey Answers)
Practical reasons (P)	P1	Convenience	Close to home; good working hours; good salary
	P2	Job opening	There was a job opening; I was asked by the workplace; it was mere fate
	P3	Continuation of internship	I gained experience through my internship and then kept on working here
	P4	Continuation from other profession	I worked here before as a nurse; I worked in this organization as a social worker; before becoming a music therapist, I worked with people with functional diversity
Connection (C)	C1	Connection out of interest	The population interests me; I have always been interested in this population; I am writing my master’s degree thesis on this population; I am curious about this population; I am interested in the reactions of these clients
	C2	Emotional connection	I like/love it; I fell in love with this population; This clientele is close to me, I can feel them and their needs; There is a good personal fit with this clientele; I’m good at it
	C3	Connection of clients to music	I see great potential in using music with this population; I had a feeling that music could be especially beneficial for this clientele; music is physically and mentally significant to them
	C4	Personal connection	This has always been my passion; personally, this population is very close to me; my son had such problems and I was looking for ways to treat him; I myself had postpartum depression and music really helped me
Innovation (I)	I	Innovative work with undeveloped field or underprivileged population	This clientele deserves more attention; this population was neglected; they were not getting enough attention and care

**Table 2:** Percentage of times that different reasons for working with clientele were mentioned: categories, subcategories in entire sample and according to country.

		Austria	Czech R.	Germany	Spain	Switzerland	Israel	Entire Sample
P1	Convenience	2	1	4	0	3	3	2
P2	Job opening	29	23	23	29	25	17	22
P3	Cont. internship	17	0	2	9	10	14	9
P4	Cont. other profession	5	22	4	14	14	3	9
C1	Connection—interest	21	15	26	14	19	11	16
C2	Connection—emotion	21	22	23	17	17	38	27
C3	Connection—music	0	2	8	3	4	4	4
C4	Connection—personal	2	4	4	8	7	6	6
I	Innovation	2	10	6	7	1	3	4
P	Total	53	46	33	52	52	37	42
C	Total	44	43	61	41	47	59	53
I	Total	2	10	6	7	1	3	4
	Total	100 *	100 *	100	100 *	100	100 *	100 *

* Rounded off to 100.

**Table 3:** Percentage of times that different reasons for working with clientele were mentioned: categories, subcategories in entire sample and according to gender (female and male music therapists).

		Female	Male	Entire Sample
P1	Convenience	2	4	2
P2	Job opening	22	23	22
P3	Cont. internship	10	6	9
P4	Cont. other profession	8	11	9
C1	Connection—interest	14	24	16
C2	Connection—emotion	29	19	27
C3	Connection—music	4	6	4
C4	Connection—personal	7	3	6
I	Innovation	4	4	4
P	Total	42	44	42
C	Total	54	52	53
I	Total	4	4	4
	Total	100	100	100

**Table 4:** Percentage of times that different reasons for working with clientele were mentioned: categories, subcategories in entire sample and according to work experience (novel, experienced, and very experienced music therapists).

		Novel Music Therapists (NMT)	Experienced Music Therapists (EMT)	Very Experienced Music Therapists (VEMT)	Entire Sample
P1	Convenience	2	2	3	2
P2	Job opening	22	25	21	22
P3	Cont. internship	13	10	4	9
P4	Cont. other profession	12	10	3	9
C1	Connection—interest	11	13	25	16
C2	Connection—emotion	26	28	26	27
C3	Connection—music	3	2	7	4
C4	Connection—personal	7	6	6	6
I	Innovation	4	5	4	4
P	Total	49	47	31	42
C	Total	47	49	64	53
I	Total	4	5	4	4
	Total	100	100 *	100 *	100

* Rounded off to 100.

**Table 5:** Percentage of times that different reasons for working with a clientele were mentioned: categories, subcategories in entire sample and according to age group of population participants work with (children, adolescents, adults, and elderly people).

		Children	Adolescents	Adults	Elderly	Entire Sample
P1	Convenience	3	3	4	1	2
P2	Job opening	21	19	23	28	22
P3	Cont. internship	9	7	12	6	9
P4	Cont. other profession	9	4	11	6	9
C1	Connection—interest	13	14	18	16	16
C2	Connection—emotion	30	38	20	27	27
C3	Connection—music	5	4	2	4	4
C4	Connection—personal	4	7	6	7	6
I	Innovation	5	5	5	4	4
P	Total	42	33	50	41	42
C	Total	52	63	46	54	53
I	Total	5	5	4	4	4
	Total	100 *	100 *	100	100 *	100

* Rounded off to 100.
